# Challenges in Physiological Phenotyping of hiPSC-Derived Neurons: From 2D Cultures to 3D Brain Organoids

**DOI:** 10.3389/fcell.2020.00797

**Published:** 2020-08-26

**Authors:** Pedro Mateos-Aparicio, Sabina A. Bello, Antonio Rodríguez-Moreno

**Affiliations:** Laboratory of Cellular Neuroscience and Plasticity, Department of Physiology, Anatomy and Cell Biology, Universidad Pablo de Olavide, Seville, Spain

**Keywords:** hiPSC, cellular cultures, organoids, neurons, astrocytes, plasticity, patch-clamp, MEA

## Abstract

Neurons derived from human induced pluripotent stem cells (hiPSC-derived neurons) offer novel opportunities for the development of preclinical models of human neurodegenerative diseases (NDDs). Recent advances in the past few years have increased substantially the potential of these techniques and have uncovered new challenges that the field is facing. Here, we outline and discuss challenges related to the functional characterization of hiPSC-derived neurons and propose ways to overcome current difficulties. In particular, the enormous variability among studies in the electrical properties of hiPSC-derived neurons and broad differences in cell maturation are factors that impair reproducibility. Furthermore, we discuss how the use of 3D brain organoids are of help in resolving some difficulties posed by 2D cultures. Finally, we elaborate on recent and future advances that may help to overcome the discussed challenges and speed-up progress in the field.

## Introduction

The field of stem cell biology has expanded rapidly over the past few years, bringing exciting technical advances as well as new avenues for treatment of human neurodegenerative diseases (NDDs). Neurons derived from human induced pluripotent stem cells (hiPSC-derived neurons) offer novel opportunities for the development of preclinical models of human NDDs such as Alzheimer’s disease (Lee et al., 2016; Mungenast et al., 2016), Parkinson’s disease (Byers et al., 2012; Fernández-Santiago et al., 2015), Huntington’s disease (Nekrasov et al., 2016), epilepsy (Parent and Anderson, 2015), schizophrenia (Wen et al., 2014; Wright et al., 2014), amyotrophic lateral sclerosis (ALS) (Chestkov et al., 2014), and autism spectrum disorders (ASD) (Darville et al., 2016; Vitrac and Cloëz-Tayarani, 2018; Drakulic et al., 2020). Since hiPSC-derived neurons contain the genetic information of the patient, they represent an invaluable resource to study diseases with strong genetic component. In addition, they offer the possibility of studying disease development and cell function in living human neurons (Dolmetsch and Geschwind, 2011).

Intense research efforts devoted to the optimization of a methodology to obtain functional neurons from hiPSCs have yielded a vast diversity of hiPSC-derived neurons from many laboratories across the world (Rhee et al., 2019). This diversity of neuronal types comes at a price of a high variability and heterogeneity in neuronal electrical properties and developmental stage, hampering reproducibility (Volpato and Webber, 2020). In addition, the use of inconsistent and variable criteria to validate hiPSC-derived neurons and incomplete functional and physiological characterization (Xie et al., 2018) add further difficulties to obtain reproducible results. Imaging techniques showing the morphology of the cells, immunohistochemistry assays showing the expression of neuronal markers and gene expression analysis of hiPSCs are necessary but not enough to confirm a neuronal phenotype. Electrophysiological and imaging techniques must be combined to describe single-cell intrinsic electrical properties, connectivity, or network activity to better describe and validate the neuronal phenotype of hiPSCs and their degree of maturation. Therefore, as the field evolves, it is becoming evident the need for standardizing and unifying the functional characterization of hiPSC-derived neurons.

In this Perspective, we will outline and discuss several challenges regarding functional phenotyping of hiPSC-derived neurons in 2D and 3D cultures. We will also highlight the problem of the developmental stage of hiPSC-derived neurons and its relevance in disease modeling. Finally, we provide our views on how some of these challenges may be overcome based on new technologies and strategies that are being implemented nowadays.

## The Challenge of Unifying Functional Measurements of hiPSC-Derived Neurons

**FIGURE 1:**
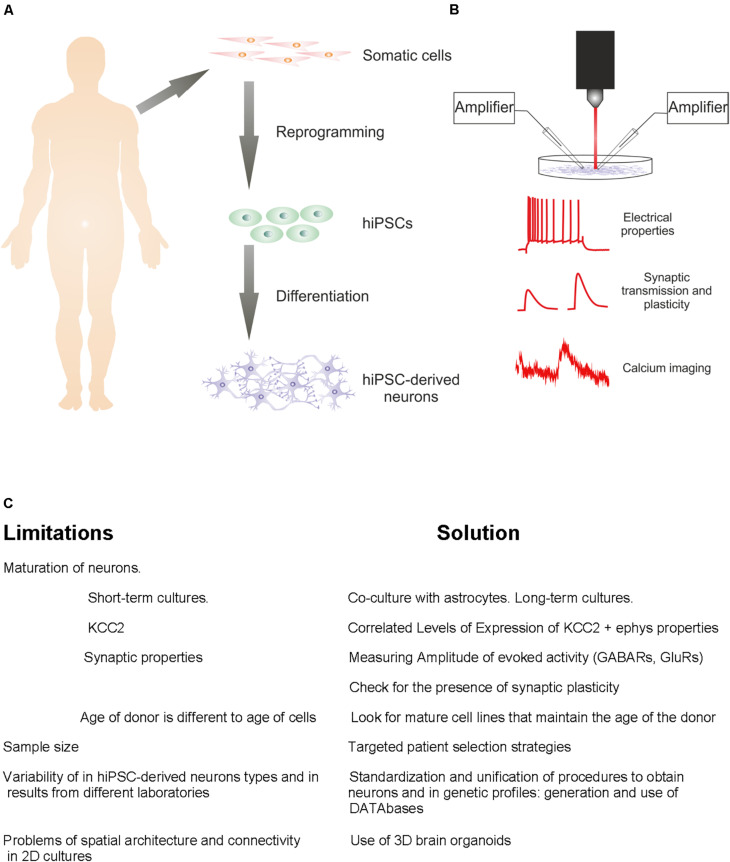
From patient to the study of hiPSC-derived neurons. **(A)** Diagram of standard steps required to convert somatic cells from diseased or healthy individuals into hiPSC-derived neurons. **(B)** Representation of electrophysiological approaches for functional characterization of hiPSC-derived neuronal characteristics. **(C)** Challenges to be resolved and solutions.

Neurons are electrical entities, as such; electrophysiological methods represent the gold standard to measure functional properties from neurons cultured in a dish to *in vivo* behaving animals (Figures 1A,B). Currently, whole-cell patch-clamp recordings of hiPSC-derived neurons are routinely performed in many laboratories worldwide and provide accurate measurements of the intrinsic properties of these cells. Relevant parameters to study the degree of differentiation of hiPSC lines such as development of stable resting membrane potential, input resistance (R_i_), membrane capacitance (C_m_), and action potential characteristics can be studied measuring the voltage response of the cell to injected hyperpolarizing or depolarizing current pulses in current-clamp mode. In addition, sodium currents underlying action potential and their kinetics can be studied under voltage-clamp mode by applying depolarizing voltage steps. Furthermore, the specific firing pattern in response to 0.5–1 s long depolarizing current pulses is a widely used criterion to classify neuronal cell types. Further analysis of active properties can be expanded by measuring ionic currents under different voltage clamp protocols, to test for the presence of specific sets of functional ion channels. In addition, the analysis of neuronal connectivity within a culture can be performed using electrophysiological techniques such as voltage and current clamp recordings that allow for detection of postsynaptic currents and potentials. As neurons mature, they contact each other following random or specific patterns, giving rise to spontaneous network activity. Impaired network activity is a hallmark of several NDDs like schizophrenia or Alzheimer’s disease.

Synaptic transmission requires the presence of functional neurotransmitter receptors. This can be measured by recording spontaneous postsynaptic currents with fast rise time and slow decay phase reflecting dynamics of presynaptic release and biophysical properties of postsynaptic receptors (Yang et al., 2011). Additionally, the pharmacological blockade of glutamate or GABA receptors during spontaneous or evoked activity can tease apart the amount of excitatory and inhibitory activity received by a single neuron (Yang et al., 2011; Espuny-Camacho et al., 2013; Liu et al., 2013; Belinsky et al., 2014; Prè et al., 2014; Wen et al., 2014; Kang et al., 2017; Xie et al., 2018; Prius-Mengual et al., 2019). Such approach also allows for the evaluation of the excitatory/inhibitory (E/I) ratio in cultured hiPSC-derived neurons. Balanced E/I ratio in cortical neurons is a key feature during information processing in mammalian neocortex (Shu et al., 2003; Haider et al., 2006; Yizhar et al., 2011; Xue et al., 2014). Furthermore, imbalanced E/I ratio has been found in multiple disorders such as epilepsy (Fritschy, 2008), fragile X syndrome (Gibson et al., 2008), autism (Nelson and Valakh, 2015) or schizophrenia (Selten et al., 2018). A fundamental property of synaptic transmission is the ability of neurons to modify the strength and efficacy of their synapses depending on intrinsic or extrinsic stimuli, a phenomenon called synaptic plasticity (commented in Mateos-Aparicio and Rodríguez-Moreno, 2019). The activity of synapses can be facilitated or depressed within different timescales, referred as short-term plasticity (lasting from milliseconds to minutes) and long-term plasticity (lasting from minutes to hours, days or weeks). Plasticity forms such as spike-timing dependent plasticity (STDP), an activity-dependent type of plasticity known to be important for circuit remodeling during development (Caporale and Dan, 2008; Markram et al., 2011), can be produced by distinct pre- and postsynaptic mechanisms (Rodríguez-Moreno and Paulsen, 2008; Banerjee et al., 2009, 2014; Rodríguez-Moreno et al., 2011, 2013; Andrade-Talavera et al., 2016) and vary their sign at distinct developmental stages (Hensch, 2004; Pérez-Rodríguez et al., 2019). Since STDP is a relevant mechanism during circuit formation that can be readily studied in neuronal cultures (Debanne et al., 2008), hiPSC-derived neuronal cultures offer an interesting platform to study synaptic plasticity in *in vitro* models of NDDs. Therefore, the analysis of synaptic transmission and plasticity must be a key step in modeling NDDs *in vitro*.

Extracellular recordings performed with multielectrode arrays (MEAs) can capture the spatiotemporal properties of overall synaptic transmission and plasticity in hiEPSC-derived neuronal cultures (Amin et al., 2016; Xu et al., 2017). This type of preparation can also provide important information about the frequency properties of network oscillatory activity in the culture, a main feature of network activity in the brain.

Patch-clamp or extracellular MEA recordings can be combined with Ca^2+^ imaging techniques to study Ca^2+^ regulation in hiPSC-derived neuronal cultures (Belinsky et al., 2014). At single cell level, membrane-impermeable fluorescent Ca^2+^ indicators can be loaded through the patch pipette and subcellular rises in cytosolic Ca^2+^ are recorded. This assay is useful to track the subcellular distribution, dynamics and properties of Ca^2+^ processes within a single hiPSC-derived neuron. As synaptic plasticity is dependent on Ca^2+^ levels (reviewed in Mateos-Aparicio and Rodríguez-Moreno, 2020), the role of Ca^2+^ in plasticity may also be determined. At the network level, multisite Ca^2+^ imaging can be performed by extracellular loading of membrane-permeable fluorescent Ca^2+^ indicators, which will be subsequently taken up by surrounding cells. Multisite Ca^2+^ imaging provides information about network Ca^2+^ oscillations and overall Ca^2+^ regulation in hiPSC-derived neuronal cultures. Recently, a novel strategy using all-optical electrophysiology has been successfully applied to characterize hiPSC-derived motor neurons in a model of ALS (Kiskinis et al., 2018). The main advantage of all-optical electrophysiology is that it captures action potential statistics and properties with higher throughput than patch clamp experiments. However, all-optical electrophysiology at the moment has lower resolution and higher noise level than patch clamp techniques, so this approach is less useful to study sub threshold events or absolute voltage values.

All methods listed above are useful for the characterization of functional properties of hiPSC-derived neurons. This is important in regard of NDDs, since most of them manifest impairment in single-cell or network activity derived from impaired functional properties. In fact, the analysis of calcium signals proved successful to discriminate schizophrenia from autism models (Grunwald et al., 2019). Since hiPSC-derived neurons are obtained from human patients, a disadvantage of using these cells is a high variability in results and availability of limited sample size at the same time, which on the other hand emphasizes the need for standardization of methods. In this regard, it has been suggested the implementation of patient selection strategies that would reduce heterogeneity. For example, in schizophrenia studies, following targeted patient selection strategies will reduce the number of non-specific phenotypes, maximizing the probability of finding causal pathways (Hoekstra et al., 2017). Also, variability of techniques in different studies makes hard to find a study applying a systematic analysis of functional properties of hiPSC-derived neurons in a culture. We suggest that efforts must be directed toward unifying and standardize techniques among studies so complete and consistent functional properties of different types of neurons can be described and compared depending on the specific needs. The creation of databases might be a step forward in this direction.

## The Maturation Problem: Are hiPSC-Derived Neurons Comparable to Human Neurons From Adult Individuals?

It is clear that patient-specific iPSCs can differentiate into neurons which can be further developed to achieve electrophysiological mature properties (Xie et al., 2018). However, the criteria defining “functional maturity” are typically vaguely described and vary between studies, so it remains unclear whether functional hiPSC-derived neurons are functionally comparable to native neurons both from control individuals and patients. In general, *in vitro* disease modeling techniques use short-term cultures which fall short of producing really mature hiPSC-derived neurons. To overcome this limitation, new approaches such as co-culture with astrocytes (Tang et al., 2013), prolonged neural differentiation (Paavilainen et al., 2018), or long-term cultures (Odawara et al., 2016; Lam et al., 2017) have proven enhanced neural maturation. The developmental stage of cells is not trivial in NDDs modeling, for example, insights obtained using cultured hiPSC-derived cell lines of Alzheimer’s disease models, need to be taken with caution since Alzheimer’s disease typically develops over the age of 70 (Penney et al., 2020).

One open question is whether donor species or age affects the degree of maturation of cultured iPSC-derived neurons. IPSC-derived neurons from mice develop their electrophysiological properties faster than those from humans *in vitro*, however, when immature hiPSC-derived neurons are transplanted into living mouse brain, they follow the developmental program of the host species (Yin et al., 2019). In general, immature neurons and hiPSC-derived neurons show depolarized resting membrane potential (>−50 mV), high R_i_, low C_m_, and low amplitude, slow kinetics, and depolarized threshold action potentials. As the cell matures, passive and active properties resemble more to those measured in primary neuron cultures, for example resting membrane potential hyperpolarizes below −50 or −60 mV and Cm increases reflecting larger membrane surface, R_i_ also decreases <1 Gohm mainly due to increased membrane surface and higher density of ion channels, action potential kinetics become faster and regenerative allowing for repetitive firing, and amplitude increases (Yang et al., 2011; Xie et al., 2018). Furthermore, a reduction of AP half-width (<3 ms) is observed (Prè et al., 2014; Bardy et al., 2016; Gunhanlar et al., 2017; Fink and Levine, 2018). In addition, synaptic properties vary according to their functional maturation state. The analysis of spontaneous synaptic activity in hiPSC-derived neuronal cultures is also used as a tool for evaluating the maturational state of the network. As the *in vitro* network matures, measurements such as the frequency of spontaneous firing or synaptic responses increase their pharmacological sensitivity to agonists/antagonists of GABA_A_, AMPA, NMDA, and KA receptors (Odawara et al., 2016). Importantly, it has been recently argued that the analysis of miniature synaptic responses, commonly used as evidence for synaptic maturation, fails to predict synaptic maturity since its frequency does not correlate with the synchronicity of evoked transmission (Meijer et al., 2019). Therefore, in order to properly assess the degree of maturation of synaptic function, conclusions should be based on the analysis of evoked synaptic transmission rather than miniature synaptic events. In addition to this, the expression of synaptic plasticity, which is typically poorly evaluated in studies using hiPSC-derived neurons, must be more thoroughly assessed (Meijer et al., 2019).

The neuron-specific membrane K^+^-Cl^–^ cotransporter 2 (KCC2) is the main responsible for setting low intracellular Cl^–^ concentration during development. Upregulation of KCC2 expression during development is responsible for the switch from excitatory to inhibitory GABAergic actions (Kaila et al., 2014). Defects in KCC2 expression levels have been found in several disorders including epilepsy (Chen et al., 2017) or neuropathic pain (Coull et al., 2005). In general, KCC2 expression is linked to neuronal maturation (Rivera et al., 1999). In animal models such as rodents, KCC2 is expressed early before birth (Rivera et al., 1999), however, upregulation of KCC2 expression occurs during the first three postnatal weeks, starting by the time of birth, with a steep increase during the first postnatal week (Kovacs et al., 2014). Some studies in humans report a negligible KCC2 expression in perinatal human brain, following upregulation during the early postnatal period, reaching a plateau after the first year of life (Dzhala et al., 2005; Jansen et al., 2010). However, a systematic analysis of KCC2 expression in humans recently reported that, in the human neocortex, KCC2 expression begins during mid-fetal period and reaches mature levels during the first 6 postnatal months (Sedmak et al., 2016). Therefore, it seems that KCC2 upregulation takes longer in human brains compared to rodent brains.

These findings suggest that KCC2 expression must be taken into account when analyzing the maturation state of hiPSC-derived neurons. It could well be that KCC2 upregulation in human neurons *in vitro* occurs faster than *in vivo*, making maturational state of hiPSC-derived neurons more comparable to miPSC-derived neurons *in vitro*. Also, hiPSC-derived neurons from Rett syndrome patients showed significant deficits in KCC2 expression and therefore a delayed functional GABAergic switch from excitation to inhibition (Tang et al., 2016). Furthermore, overexpression of KCC2 in these neurons rescued functional GABA deficits, suggesting the restoration of KCC2 function as a strategy for potential treatment of Rett syndrome and perhaps other autistic-like disorders (Tang et al., 2016). In conclusion, the KCC2 expression pattern and temporal profile must be assessed together with the electrophysiological properties of hiPSC-derived neurons to properly analyze their functional maturation state *in vitro* (Figure 1C).

## Toward the Brain *in vitro*: Functional Studies in Brain Organoids

The challenges of spatial architecture, connectivity and distribution present in conventional 2D cultures of hiPSC-derived neurons are being resolved with the rise of 3D brain organoids (Cakir et al., 2019; Qian et al., 2019). When hiPSC-derived neurons are grown in 3D aggregates instead of in direct contact with a flat surface, they are capable of self-organize and differentiate in organized, patterned structures that resemble the fetal human brain. From a structural point of view, brain organoids are much smaller than human cerebral cortex, but they recapitulate important aspects of human cortical architecture like neuronal layer patterning or neural lineage cell types. In light of the challenges discussed above in 2D hiPSC-derived neuron cultures, functional studies in 3D brain organoids by means of patch-clamp recordings showed a high variability and different maturational stages of cells, although this variability is constrained within a range of immature functional phenotypes. Experiments in more mature organoids, although still immature, revealed some degree of maturation, in the form of robust excitatory and inhibitory currents as well as trains of action potentials upon current injection (Paşca and Sloan, 2015), indicating certain degree of maturation over time. Extracellular multi-electrode recordings and photostimulation in organoids developed over more than 9 months have shown burst-like firing activity and relatively more mature functional properties (Quadrato et al., 2017). Overall, although hiPSC-derived neurons in brain organoids are able to mature over time (Qian et al., 2016), literature is consistent with the idea that neurons in 3D cultures are immature and comparable to neurons in embryonic stages (Paşca and Sloan, 2015; Qian et al., 2016, 2019; Birey et al., 2017). While these methods are excellent to model NDDs that show alterations during development, they fall short in diseases like Alzheimer’s disease in which alterations manifest much later in time. Recent developments, like the so-called assembloids, which result from the fusion of previously generated region-specific organoids, or from a mixture of cells of different lineages with biomaterials or cells with organization capabilities, offer the potential to develop more complex models of inter-regional interactions that can better approach network activity in different NDDs (Paşca, 2018).

## Future Perspectives

We have discussed several challenges that the stem cell research field is facing in light of recent newly developed iPSC technologies like the use of hiPSC-derived neurons in 2D and 3D cultures. In the short-term future, it will be necessary to standardize and unify the plethora of hiPSC-derived neuron lines and their methods of obtention, functional properties, genetic profiles, pharmacology, among others. At this point, a new issue arises, the organization of the amount of information generated in different laboratories using different techniques. A way to solve this problem is the creation of databases containing morphological, genetic, protein expression, biophysical and electrophysiological properties, combined with differentiation protocols for the generation of specific hiPSC-derived neurons. In this regard, existing efforts in current neuroscience oriented to improve neuronal classification in animal models can be helpful to create similar platforms related to hiPSCs. In particular, examples of data sharing initiatives which may be of interest for the stem cell research field are the Allen Brain Institute Cell Types Database, which is producing massive quantities of data on neuronal cell types (Stockton and Santamaria, 2017), the Neurodata Without Borders consortium led by the Kavli Foundation with the goal of develop open-source data format of cellular neurophysiological data (Teeters et al., 2015), the Neuroelectro project which has the objective of compiling and organizing published data about electrical properties of neurons (Tripathy et al., 2014), or the ModelDB database which compiles and organizes published biophysical model of neurons (Hines et al., 2004). Such initiatives may serve as a model for the stem cell research field to share data and organize information for better reproducibility. Such databases can be analyzed by using machine-learning based methods to help identify which cell line is best to model a specific NDD, thereby saving time and improving reproducibility of results. Machine-learning techniques are already in use and have proven effective in improving reproducibility by helping automatic detection of reprogrammed iPSC colonies (Fan et al., 2017), for example.

In order to overcome the challenge of maturation of hiPSC-derived neurons, the creation of mature cell lines that maintain the age of the donor or methods for accelerated aging in 2D and 3D cultures are an exciting possibility that may provide a new paradigm in the field (Mertens et al., 2018; Traxler and Edenhofer, 2019). Finally, in the recent years the functional properties of living human neurons in brain slices have been elucidated. Human cortical neurons show remarkable properties and computational capabilities that differ significantly from those observed in rodents (Mansvelder et al., 2019; Gidon et al., 2020). For example, recently it was shown that human pyramidal neurons in the neocortex display previously unknown classes of dendritic action potentials, making their activity much more complex than previously thought (Gidon et al., 2020). It would be of great interest in the future to explore dendritic functional properties of hiPSC-derived neurons to compare with regular human neurons and confirm their similarities in the information processing.

## Author Contributions

All authors listed have made a substantial, direct and intellectual contribution to the work, and approved it for publication.

## Conflict of Interest

The authors declare that the research was conducted in the absence of any commercial or financial relationships that could be construed as a potential conflict of interest.
